# Nucleic Acids for Ultra-Sensitive Protein Detection

**DOI:** 10.3390/s130101353

**Published:** 2013-01-21

**Authors:** Kris P. F. Janssen, Karel Knez, Dragana Spasic, Jeroen Lammertyn

**Affiliations:** MeBioS Biosensor Group, Faculteit Bio-Ingenieurswetenschappen, KU Leuven, Willem De Croylaan, Leuven, Belgium; E-Mails: kris.janssen@biw.kuleuven.be (K.P.F.J.); karel.knez@biw.kuleuven.be (K.K.); dragana.spasic@biw.kuleuven.be (D.S.)

**Keywords:** nucleic acid, biosensor, protein, biomarker, PCR, aptamer

## Abstract

Major advancements in molecular biology and clinical diagnostics cannot be brought about strictly through the use of genomics based methods. Improved methods for protein detection and proteomic screening are an absolute necessity to complement to wealth of information offered by novel, high-throughput sequencing technologies. Only then will it be possible to advance insights into clinical processes and to characterize the importance of specific protein biomarkers for disease detection or the realization of “personalized medicine”. Currently however, large-scale proteomic information is still not as easily obtained as its genomic counterpart, mainly because traditional antibody-based technologies struggle to meet the stringent sensitivity and throughput requirements that are required whereas mass-spectrometry based methods might be burdened by significant costs involved. However, recent years have seen the development of new biodetection strategies linking nucleic acids with existing antibody technology or replacing antibodies with oligonucleotide recognition elements altogether. These advancements have unlocked many new strategies to lower detection limits and dramatically increase throughput of protein detection assays. In this review, an overview of these new strategies will be given.

## 1. Introduction

In biological systems, information is passed down from the deoxyribonucleic acid (DNA) to the protein level with RNA as an intermediate. This is known as “the central dogma of molecular biology” [1] and for biological systems there seems to be no escaping this simple fact. In the field of biosensor research however, recent years have seen an ever-increasing trend towards the use of deoxyribonucleic acid (DNA)-and ribonucleic acid (RNA) oligonucleotides as mediators in the detection and quantification of protein biomarkers, effectively resulting in a reversal of the flow of information between nucleic acids and proteins.

The causes for this reversal can be traced back to the realization that major advancements in molecular biology and clinical diagnostics cannot be brought about strictly through the use of modern, next generation whole genome sequencing technologies. Indeed, even though the genome essentially offers a blueprint for all the building blocks of life, there is no strict, one-to-one relationship between the genotype and the phenotype of an individual [2]. As such, the predictive value of all this newly available genetic information with regard to e.g., disease diagnostics or even disease prediction is severely limited [2], and large scale availability of proteomic information to complement the genomic data remains indispensable [2,3]. The proteomes of even “simple” single cell organisms can be highly complex however [4] and the human proteome is a few orders of magnitude larger still, showing a very large dynamic range, easily surpassing 10 orders of magnitude [5]. Obtaining high resolution, high coverage data is therefore very challenging.

**Figure 1.**
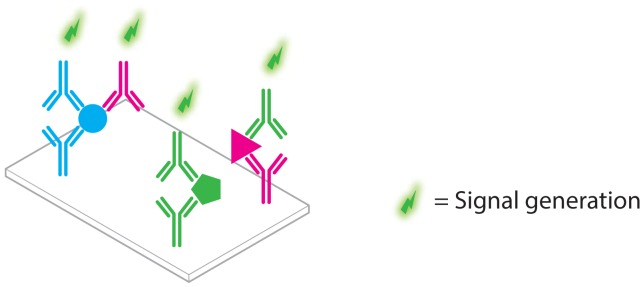
When trying to multiplex a typical ELISA, the risk of false positive detection due to immuno reagent cross-talk increases rapidly with increasing amounts of targeted analytes.

For decades, immunoassays have been the primary source of proteomic information in both life science research and clinical diagnostics. In their seminal work, Yalow and Berson [6] were the first to report on a radio-immunoassay for the direct detection of insulin levels in blood. Since then, the development of the enzyme-linked immunosorbent assay (ELISA) [7] has negated the need to use potentially harmful radio-isotopes in bioassays. Additionally, enzyme-linked immunosorbent assay (ELISA) offers many more advantages such as its ease-of-use, flexibility and relatively low cost. Unsurprisingly, the success of enzyme-linked immunosorbent assay (ELISA) is evidenced by thousands of studies published each year utilizing the technology [8,9]. Even with more recent developments in the field of mass spectroscopic analysis [10,11], ELISA remains one of the benchmark technologies in proteomics applications. However, ELISA is not without its share of drawbacks and limitations, especially when it comes to analyzing large numbers of proteins in a multiplexed fashion with only limited total amounts of sample. Indeed, ELISA is often performed in a well plate format and if sample volumes are limited, multiplexing of an assay naturally implies that multiple antibody pairs should be combined in a single well (Figure 1), which will inevitably lead to cross-reactivity [12].

Furthermore, the fact that ELISA is usually dependent on fluorogenic or chromogenic substrates for actual signal generation will also severely limit the number of simultaneous detection events that can be achieved, since the number of distinct dyes as well as the number of detection channels in typical readout instruments is usually limited. In this respect, nucleic acid mediated protein assays, where a specific deoxyribonucleic acid (DNA) sequence is coupled to or even wholly constitutes the affinity reagent, might offer some unique benefits [12]:

- DNA and RNA both adhere to simple complementarity rules, which cater the easy design of specific interactions between different probes and affinity reagents
- Oligonucleotides can be easily synthesized and chemically modified at scale, affording flexibility in assay design
- Oligonucleotides can be processed with a large and versatile enzymatic “toolbox” that enables the facile amplification of oligonucleotide material
- Specific sequences inherently carry information that can be used for direct “barcoding” of the reagents and/or analytes to which they are coupled

**Figure 2.**
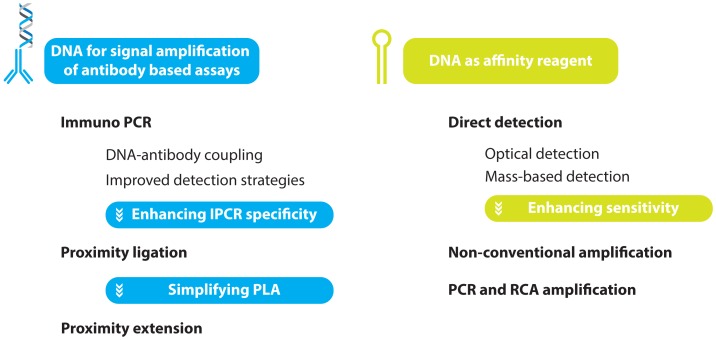
A schematic overview of the topics under review.

In this review, an overview will be given of the most recent developments with respect to oligonucleotide mediated protein assays. These will include examples where DNA is used purely for signal amplification and multiplexing in conjunction with conventional antibody recognition elements as well as methods where the olignucleotides themselves (*i.e.,* aptamers) are the recognition elements (Figure 2).

## 2. Protein Detection Using DNA Labels

Enzymatic methods to amplify DNA have been available for a long time [13,14] and the original polymerase chain reaction (PCR) as well as its many variations are part of the standard biotechnology toolbox [15]. It is therefore not unsurprising that these methods have also been applied for signal amplification in DNA assisted protein detection.

In general, three distinct assays are known that rely on enzymatic replication of DNA labels for ultra-sensitive protein detection:

- immuno PCR (IPCR)
- proximity ligation assay (PLA)
- proximity extension assay (PEA)

**Figure 3.**
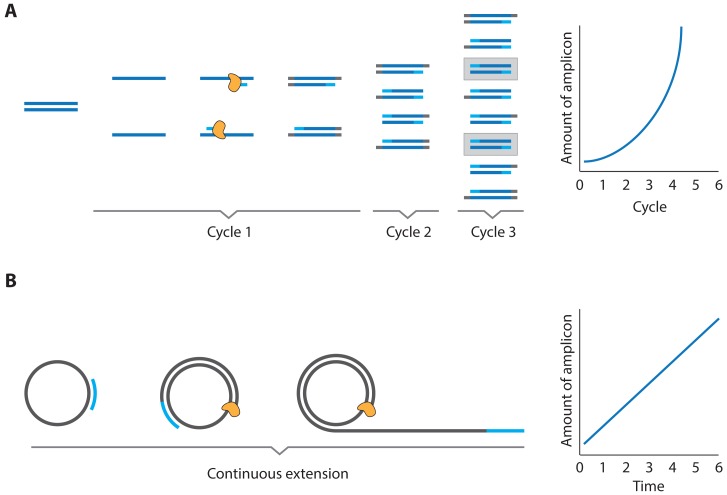
Schematic overview of (**A**) PCR, where an initial dsDNA template is first dehybridized, after which primers anneal to the ssDNA strands. These primers provide a 3′ extendable terminus that is used for polymerase synthesis of the complementary strand. Subsequent discrete cycles of dehybridization and primer extension lead to an exponential increase of amplicon. (**B**) In RCA, a primer anneals to a circular ssDNA template, often called a padlock probe, polymerase extension at the 3′ primer origin leads to a continuous synthesis of ssDNA amplicon that consists of concatenated complements of the original template, leading to a linear increase of amplicon.

These assays can be categorized further when the actual method for enzymatic amplification is considered. Two systems have been commonly used; PCR [13,14] and rolling circle amplification (RCA) [16,17], each with their own strengths and weaknesses. An overview for each of these amplification methods is given in Figure 3.

### 2.1. Immuno PCR and -RCA

**Figure 4.**
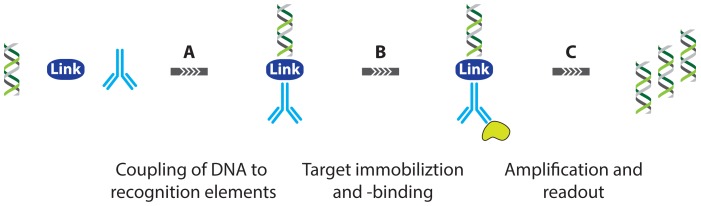
Schematic overview of IPCR where dsDNA is coupled to an antibody through the use of various linkers (**A**). Target binding (**B**) and subsequent amplification of the dsDNA label allows for sensitive quantification of the target (**C**).

The first report on immuno-PCR in literature can be attributed to Sano *et al.* [18] who used DNA-linked instead of enzyme-linked antibodies as a secondary labeling agent in an assay that is otherwise very similar to conventional ELISA. This way, signal amplification in protein detection was established, effectively leading to a limit of detection for bovine serum albumin (BSA) of 2 femtomolar, which constitutes an improvement in sensitivity by a factor of 10^5^ compared with the conventional ELISA. To achieve this goal, a recombinant protein was specifically prepared, linking protein A and streptavidin. This recombinant protein could then be used to bind to biotinylated double stranded DNA (dsDNA) while the protein A moiety could bind to a specific domain of immunoglobulin G (IgG). In this way, the DNA tagged chimera could serve as a label to the antigen-primary antibody complex. Conventional PCR amplification of the dsDNA tag then theoretically allows accurate readout of even single molecule amounts of antigen present in the sample (Figure 4).

Even though this first report of IPCR presented a great technological achievement, the assay also suffers some drawbacks that limit its more general application [19]. Indeed, the fact that a chimeric protein was used to achieve coupling between the DNA label and the primary monoclonal antibody (mAb) is one of the biggest issues as these chimeras might not always be readily available. Furthermore, Sano *et al.* [18] only demonstrated the IPCR concept using a direct assay where analyte was sorbed to the walls of the sample recipient directly, without the use of a specific capture antibody. In fact, use of such capture antibodies is not possible since the protein A moiety of the chimera would also bind to them directly, even in the absence of analyte, leading to high backgrounds and false positive results. Moreover, reliance on the initial adsorption of analyte to the solid support limits the scope of the assay since not all proteins will be readily immobilized, and even if they could, co-adsorption of matrix proteins might significantly increase background signals or even wholly prevent the analyte from being detected. Lastly, the amount of PCR amplicon was detected using slab gel electrophoresis, which somewhat limits the quantitative resolution and adds to the overall complexity and duration of the assay.

In the time since the original report, many groups have worked to address the weaknesses of IPCR by focusing on one or more of the three key aspects of the assay workflow; DNA-antibody linking, target immobilization and -binding and assay readout (Figure 4).

#### 2.1.1. Improved Methods for DNA-Antibody Coupling in IPCR

**Figure 5.**
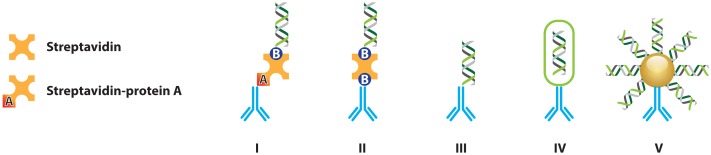
Different strategies for coupling antibodies and DNA for use in IPCR: (**I**) A Streptavidin-protein A chimera protein was used for labeling of the detection antibody with biotinylated DNA. (**II**) Universal IPCR uses biotinylated antibodies that are labeled with dsDNA through subsequent incubations with streptavidin and biotinylated dsDNA. (**III**) Direct coupling of antibodies and dsDNA can in some cases be achieved using chemical methods. (**IV**) Specialized coupling strategies exist where single- or multiple copies of labeling DNA are encapsulated inside of liposomes, supramolecular constructs or even phages. (**V**) Using readily available biofunctionalization strategies for micro- and nanoparticles, both the antibody and the labeling dsDNA can be immobilized on the surface of the particles, thus effectively achieving coupling between the two.

An obvious enhancement to the original IPCR would be to replace protein A in the originally used chimera such that the general cross-reactivity towards IgG (*i.e.*, in capture antibodies) can no longer occur. A number of the strategies reported in literature are schematically presented in Figure 5 and will be briefly addressed in the following sections.

Ren *et al.* [20] achieved antibody dsDNA coupling through a specialized antibody-streptavidin chimera, which was applied for the detection of specific antigens involved in breast cancer. By directly coupling the detection antibody to streptavidin, non-specific binding of the originally reported chimera through its protein A moiety could no longer occur. However, even though the assay marked a significant improvement over conventional ELISA, it still requires the production of highly specialized recombinant proteins and therefore it is not easily generalized. Moreover, the authors do not use a sandwich type assay where usage of distinct capture and detection antibodies would increase specificity.

**Figure 6.**
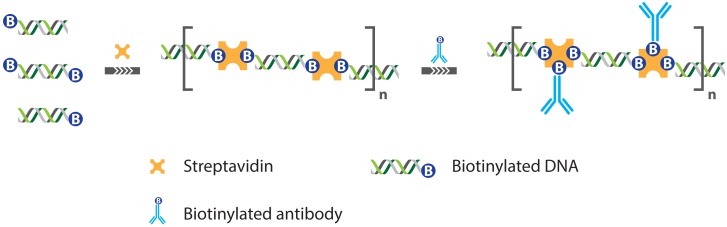
Schematic representation of self-assembled oligomeric DNA-protein complexes used in IPCR. Mono- and bis-biotinylated dsDNA was mixed with equimolar amounts of streptaidin to generate oligomeric DNA-streptavidin complexes. The resulting DNA-streptavidin networks could be functionalized by coupling with biotinylated antibodies, and after sufficient purification, these functionalized networks could be applied as reagents in IPCR [24].

Most of the research on IPCR quickly shifted focus on the use of multivalent linkers to achieve coupling of the DNA label and the antibody. One of the earliest examples of this approach came with the development of so-called “universal” IPCR where the streptavidin-protein A chimera was omitted in favor of direct streptavidin mediated coupling between biotinylated antibodies and dsDNA. Zhou *et al.* [21] could show that this approach offers a sensitivity that matches that of the originally reported IPCR with a limit of detection (LOD) of 9.6 femtomolar for the Ets1 protein, a cancer biomaker [22]. Furthermore, their approach brought with it many advantages, chiefly the fact that biotinylated antibodies are commonly used in biotechnology and therefore more readily available as well as the fact that now, successive protocol steps such as the capture of the analyte using primary antibody, incubation with a secondary antibody, labeling of the antibody with streptavidin-DNA adduct could be performed in sequence, allowing for sample washing between each step [23]. These have quickly made universal IPCR the most widely used assay format [19]. However, the requirement of multiple incubation steps will inevitably lead to incomplete formation of the antibody-streptavidin-DNA complex, which has the potential to reduce assay sensitivity, offsetting any benefits the approach might offer if sufficient care is not taken. With the aim of tackling those issues, the ability of streptavidin to bind multiple biotinylated dsDNA molecules was exploited to construct supramolecular complexes of dsDNA and streptavidin (Figure 6). Through careful control of the reagent ratios, the structure and size of these complexes could be accurately controlled, resulting in nanostructures containing unsaturated streptavidin moieties that could further be functionalized with biotinylated antibodies. The thus obtained complexes proved excellent reagents in IPCR assays and help to resolve most of the issues encountered with universal IPCR, because the supramolecular complexes could be assembled and purified before analysis such that only correctly formed complexes could be used for analysis [24]. This way, an IPCR assay was demonstrated with a 30 femtomolar limit of detection for rabbit IgG as a model analyte, which constitutes an improvement of 10^2^ over ELISA and a tenfold improvement compared with universal IPCR [24].

Even though antibodies, being proteins, are prone to denaturing, chemical treatment allowing for the direct formation of a covalent bond between the antibody and the dsDNA has nevertheless been reported [25,26]. Directly linked conjugates offer a number of advantages in IPCR as they do not require lengthy pre-incubation steps before the actual analysis can take place. Furthermore, since the dsDNA labels are irreversibly linked to their respective antibodies, there is no risk of label exchange between reagents, making it easier to design multiplexed IPCR assays without significantly compromising assay sensitivity with LODs of 2, 200 and again 200 femtomolars for hTSH, *β*-Gal and hCG protein model targets respectively [25]. Here, it is important to note that the authors attribute the higher LODs for the latter two targets to the short DNA labels used in comparison with those used by Sano *et al.* [18] and not to the covalent link. These issues could easily be mediated in an optimized assay. Even so, the reagents required to achieve the covalent link are often difficult to obtain, with different proteins presenting different functional surface groups or different distributions of these groups, each requiring different experimental conditions. On top of that, the functionalization process itself can prove lengthy, requiring multiple purification steps that can suffer from low yields. It is therefore not completely surprising that the covalent linking strategy as yet has not been as widely reported as other IPCR strategies.

More recently, encapsulation of reporter DNA in the liposomes that are functionalized with recognition elements such as antibodies or receptor proteins was shown to offer two distinct advantages; Since multiple reporter DNAs are present at each binding site, higher sensitivity can be achieved, leading to impressive limits of detection as low as 0.1 attomolar for Botulinum toxin. Furthermore, liposome encapsulated DNA is impervious to enzymatic degradation. This is useful because it on the one hand protects the reporter DNA from premature degradation due to matrix components but it also allows the experimenter to treat the sample with DNase to remove any possible background contamination [27].

**Figure 7.**
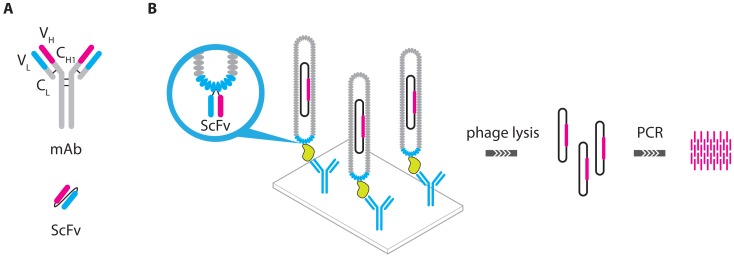
(**A**) ScFv consists of the variable regions of the heavy (V_H_) and light chains (V_L_) of whole immunoglobulins, connected with a short linker peptide. (**B**) When the encoding gene for an ScFv is inserted into the phage genome at the correct location, the ScFv will be expressed on the phage mantle, allowing the phages to selectively bind target proteins. In a phage IPCR assay, target binding can be followed by phage lysis after which the phage DNA can serve as the template for PCR amplification [28].

In another modification of the immuno-PCR, recombinant phage particles are used instead of antibody-DNA conjugates [28]. On the surface of a phage particle, a fusion protein was expressed, consisting of the variable regions of immunoglobulin heavy and light chains, connected via a short linker peptide. This single chain variable fragment (ScFv) was used as a recognition element whereas the phage DNA could subsequently be used for quantification (Figure 7).

Although the proof-of-concept work showed poorer limits of detection—approximately 200 femtomolar for a viral nucleoprotein—compared with those offered by conventional ELISA, mainly due to the more limited affinity of the ScFv, it at the very least offers the prospect of enabling the preparation of IPCR reagents using existing and relatively straightforward phage display methods [29].

**Figure 8.**
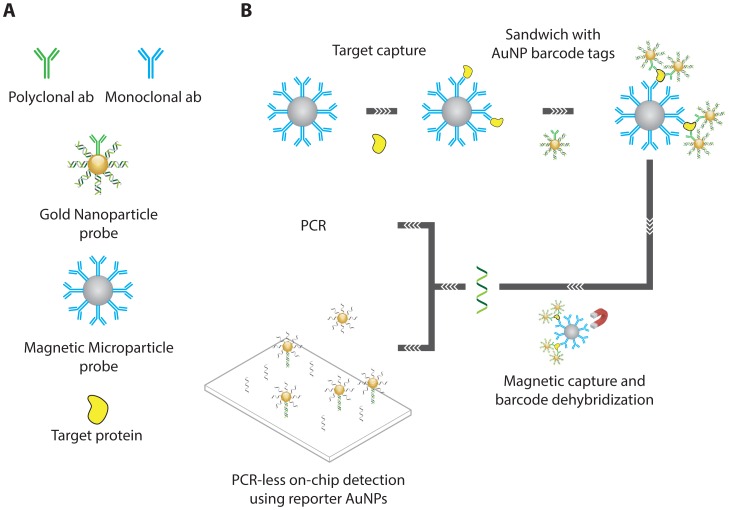
Overview of the bio-barcode assay. (A) For a specific target, mAb-functionalized MMPs are prepared, along with pAb functionalized DNA-AuNP conjugates. (B) In a typical experiment, an aqueous dispersion of MMP probes is mixed with an aqueous solution of sample. After incubation, the MMPs, having captured the analyte of interest, are concentrated using a magnet. The supernatant is removed, and the MMPs are resuspended. This process is repeated to wash the MMPs. The AuNP probes are then added to the assay solution. The AuNPs reacted with the target immobilized on the MMPs and provide DNA for signal amplification and protein identification. After sufficient washing of the MMP-AuNP sandwich, they are resuspended in pure water at elevated temperature to dehybridize barcode DNA strands from the nanoparticle probe surface. Dehybridized barcode DNA was then separated and collected from the probes with the use of the magnetic separator [30].

Nam *et al.* [30] demonstrate for the first time how short DNA oligonucleotides can be used as “barcodes” for the identification of protein targets in a homogeneous sandwich assay. As shown in Figure 8, the multi-step assay proceeds by incubation of a small sample aliquot with mAb-functionalized magnetic nanoparticles. After sufficient incubation time, the analytes will be captured on the magnetic particles, allowing them to be separated from the sample matrix and washed to remove any non-specifically adsorbed matrix components. After this initial step, the magnetic microparticle (MMP) captured analyte is incubated with mAb functionalized gold nanoparticles (AuNPs). Next to mAbs these secondary particles also carry a dense layer of short dsDNA.

In fact, special care is taken during the preparation of these secondary particles to ensure a high dsDNA to mAb ratio, which is an important factor in determining the overall assay sensitivity as each binding event of a secondary particle effectively contributes many dsDNA barcode sequences, thus providing signal amplification. After incubation with the secondary particles, the MMP, AuNP adducts are separated a second time and washed. After washing, the dsDNA barcodes are denatured and detected on a chip carrying complementary DNA to allow optical readout.

The proposed approach carries with it a number of significant advantages; Firstly, by using functionalized nanoparticles (NPs) as opposed to plate-immobilized affinity ligands, the assay avoids potential issues of diffusion limitations in the actual capturing of the analyte whilst also maintaining high MMP to analyte ratios, making the overall target binding much more efficient. Secondly, the MMPs allows pre-concentration of the analyte prior to detection as well as complete removal of interference from matrix components or non-specifically bound barcodes, which could both contribute to high background signals. Finally, as already mentioned, the use of secondary AuNPs ensures a high DNA barcode to secondary mAb ratio, which is beneficial for assay sensitivity. The authors could thus report a LOD of 3 attomolar for PSA protein.

Stoeva *et al.* [31] further show how the assay can easily be parallelized to include multiple analytes, simply by simultaneously incubating the initial sample volume with different MMP/AuNP combinations where each pair is marked by a specific barcode sequence. Here again, the approach offers the benefit of only requiring a small sample volume to effectively detect multiple analytes whereas the stringent washing steps ensure minimal cross reactivity and low background signals. The relatively easy format of the assay also enables miniaturization and automation [32].

#### 2.1.2. Improved Detection Strategies in IPCR

**Figure 9.**
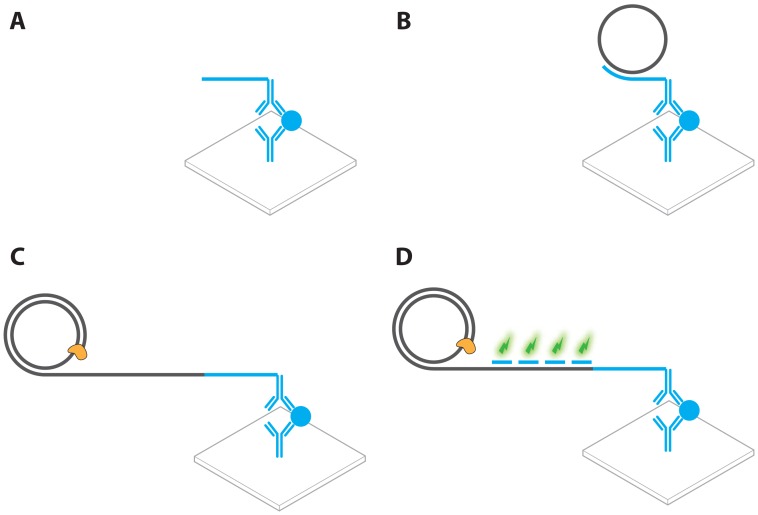
Schematic overview of the immuno-RCA assay. (**A**) DNA labeled antibodies are used in an otherwise standard sandwich immuno assay. (**B**) Hybridization of a circular DNA probe (**C**) RCA. (**D**) The amplified product is labeled through hybridization with fluorescently labeled oligonucleotides, leading to significant signal amplification.

Replacing the PCR with RCA in IPCR has been shown to offer significant benefits. Immuno RCA (IRCA) is conceptually not very different from IPCR. However, RCA of the DNA label attached to the detection antibody enables the linear amplification of a simple, circular ssDNA probe, leading to the localized production of a single, large ssDNA amplicon [33,34]. This amplicon consists of a large number of repeats, which are all the complement of the circular ssDNA. First and foremost, the accumulation of ssDNA amplicon will result in signal enhancement. Indeed, the repetitive nature of the amplicon allows for the hybridization of a large number of short, fluorescently labeled ssDNA probes, which will generate a detectable signal in the case of a binding event (Figure 9).

Secondly, the fact that RCA can be performed isothermally means that the instrumentation required for IRCA can be significantly less complex compared with IPCR and on top of that, the conditions for RCA are compatible with antibody target binding, meaning that detectable amplicon will remain localized at the site where target recognition originally occurred [35]. This is important as it opens the door to the application of IRCA towards high-throughput protein microarray assays. The fact that the amplicon stays localized means that identical fluorescent reporter probes can be used for detection, with identification of the actual target molecule resulting from the knowledge of the capture antibody present at each location. This is a huge reduction of the assay complexity over the multiplexed PCR necessary for IPCR, which would require the design of many probes in order to identify individual DNA sequences in an amplification mixture [36–39].

### 2.2. Improving the Specificity of IPCR: The Proximity Ligation Assay

**Figure 10.**
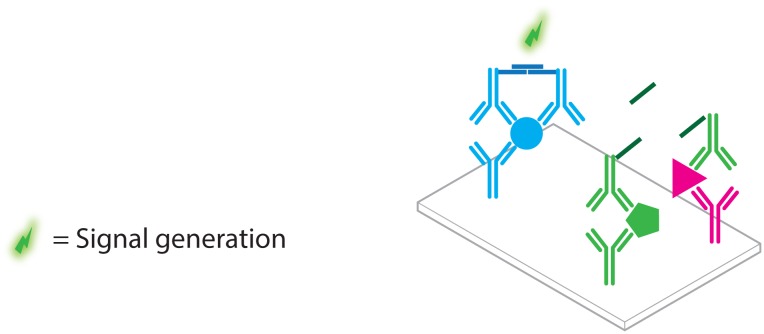
The proximity ligation assay is specifically designed to suppress cross-talk and matrix effects in IPCR.

The development of the PLA assay can be viewed as a natural evolution of IPCR. Although the format of the latter evolved to allow for ever lower background and assay specificity, there was still room for improvement. Some groups had already shown how the “homogenization”, *i.e.*, conducting the assay completely in the liquid phase, of IPCR could bring significant benefits (notably the various types bio-barcode assays, [30,40,41], *vide supra*). Even with the use of so-called magneto IPCR however, there was still opportunity for non-specific interactions between matrix components and the capture MMPs used, even after stringent MMP washing. With the PLA, developed by the Landegren group [42,43], these non-specific interactions are largely mitigated. In the PL,A the sample is incubated with two or more ssDNA functionalized affinity reagents, most often antibodies and a separate ssDNA linker oligonucleotide. The oligonucleotide tags of the affinity reagents will only be in close enough proximity to allow hybridization with the linker when target binding occurs (Figure 10). When this happens, the linker hybridized tags can be ligated and subsequent PCR amplification allows their quantification. The complete assay can occur in the liquid phase without the need of any immobilization or washing steps and since two distinct binding events are necessary for detection to occur, the specificity of the assay is drastically improved [42].

**Figure 11.**
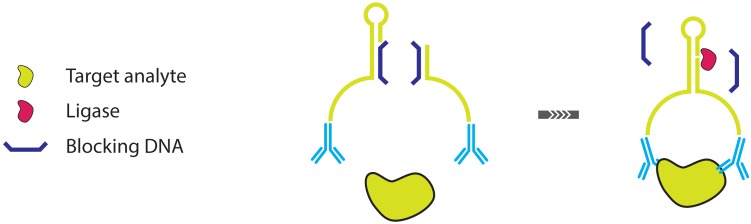
A schematic overview of the BINDA assay, an alternative format of PLA.

Zhang *et al.* [44] have recently demonstrated another interesting approach to reduce background in PLA by omitting the linker oligonucleotide and linear label sequences in favor of partially double stranded labels with hairpin structures. The rationale behind the proposed Binding Induced DNA Aplification (BINDA) assay is based on the knowledge that structural motifs can have a significant impact on the melting temperature of dsDNA [45]. When two distinct DNA labeled antibodies are not bound to their target, the melting temperature of the duplex between both labels is lower than the melting temperature between each individual label and its “blocking” oligomer, thus preventing ligation in the absence of target (Figure 11). The assay thus offers high sensitivity, with LODs down to 0.1 femtomolar for PSA, coupled with a high resilience against non-specific signal generation as evidenced by the fact that these low levels of analyte could be detected in a complex serum matrix.

PLA is a very convenient assay as the total number of process steps is essentially limited to adding and mixing of the affinity reagents followed by the addition of PCR reagents which are also available in convenient pre-mixed form [46]. Nonetheless, care should be taken exactly with the addition of the affinity reagents since it is obvious that at increased concentration of these reagents, the chance of linker hybridization even in the absence of target will increase, leading to high backgrounds. In practice, this can be addressed by improved *in silico* design of the ssDNA label and linker sequences to eliminate non-specific interactions. Shortening of the label and linker sequences in combination with ligation using thermostable ligases at elevated temperature and optimized ionic strength will also increase the specificity of hybridization. Finally, by diluting the sample after incubation of the affinity reagents and linker but before ligation, the concentration of labels and linkers can be reduced while target bound probes remain in close proximity [47].

**Figure 12.**
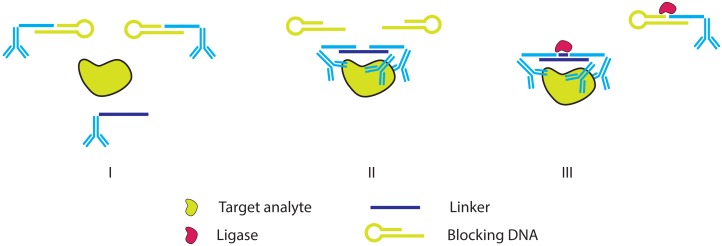
The triple PLA. (**I**) Free proximity probes are blocked using partially complementary hairpin probes. (**II**) The target protein is recognized by three proximity probes simultaneously, each targeting a different epitope. The blocking hairpins are displaced by a linker ssDNA. (**III**) Ligase is used to merge the affinity probe ssDNA moieties and linker ssDNA and unbound affinity probes are simultaneously locked with blocking hairpins. Subsequent detection of the linked probes can be achieved using qPCR [50].

Next to improvements of the basic PLA protocol, a number of more extensively modified PLA assays have also been proposed such as various solid phase formats. Here, the proximity ligated products are captured on a solid surface using a third epitope on the target protein, leading to additional possibilities for sample extraction and washing [48,49], combining the advantages of regular PLA and MMP immobilized IPCR. Alternatively, triple PLA requires simultaneous binding of three distinct affinity reagents in order to obtain a detectable DNA duplex (Figure 12, [47,50]). Recently, the combination of a surface immobilized PLA format with the triple binding concept has lead to the development of a highly parallelized ultra-sensitive protein assay [51].

Finally, replacing the linker with a padlock probe in PLA makes it possible to replace PCR with an isothermal amplification step in order to quantify ligated affinity probes. This RCA can be performed under isothermal conditions and generates macroscopic amplicon “blobs”, which makes this method ideally suited for implementation on microfluidic platforms [52].

### 2.3. Further Simplifying IPCR and PLA: The Proximity Extension Assay

**Figure 13.**
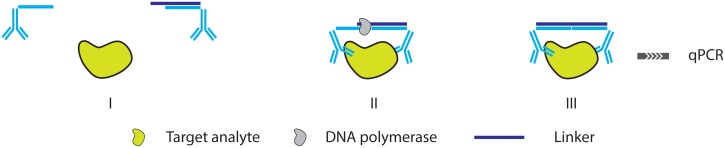
A schematic overview of the PEA assay [53].

It is possible to omit the linker hybridization and subsequent ligation steps in the PLA, which can help to simplify the overall assay, maximizing assay efficiency without losing its other advantages [53]. With PEA, one of the affinity reagents is conjugated with a dsDNA label instead of ssDNA such as with PLA and this dsDNA features a 3′ overhang. Proximity binding of both reagents, however, will convert this overhang into a priming site for DNA replication (Figure 13). Lundberg *et al.* [53] show how the usage of exonuclease enabled polymerase for probe extension can contribute significantly to the suppression of assay noise. Reduced noise, coupled with the reduced complexity of the PEA, enables LODs between 0.1 and 1 pM for multiple analytes, even in multiplexed assays and for samples contained in a complex serum matrix. Other variations of PEA exist, but each time, a 3′ extensible position is created exclusively upon target induced proximity binding [54,55].

## 3. Aptamer Based Ultra-Sensitive Protein Asays

Aptamers are versatile oligonucleotide biorecognition elements whose target selectivity and affinity can rival antibodies. Not only can they serve to replace antibodies because of their more appealing physicochemical properties, their inherent base pairing ability also allows them to specifically interact with linker- or beacon- oligonucleotides. Moreover, their secondary structure, responsible for their target binding abilities, can even be influenced through interactions with other oligonucleotides. Likewise, the same enzymatic methods for amplification and the detection strategies that rely on them, as discussed in previous paragraphs, are also applicable to aptamers. Aptamers can thus be deemed a more versatile type of antibody opening up ways for innovative detection and signal amplification schemes in protein detection. However, application of aptamers without a basic knowledge of their biochemistry or technical requirements can cause serious analytical difficulties [56].

Many excellent reviews exist on the use of aptamers in biosensing [56,57] and providing an exhaustive overview of aptamer-based biosensors would prove well beyond the scope of this review. Here, a selection of the most notable examples will be reviewed.

### 3.1. Non-Amplified Systems

Most aptamer-based biosensors rely on the tagging or labeling of the aptamer with a reporter moiety. This reporter can be anything ranging from an organic dye for colorimetric detection, a fluorophore, various electrochemical mediators, enzymes or nanoparticles that ultimately allow optical, electrochemical or mass based detection of the aptamers or the target binding event.

#### 3.1.1. Optical Aptasensing Assays

Dye- or fluorescent labeling of DNA is relatively straightforward and many commercial reagents exist for this purpose. Moreover, equipment for colorimetric and fluorescence based detection (e.g., various plate reader devices and spectrophotometers) is part of the standard equipment in most laboratories and research centers. It is therefore not surprising that optical aptamer based assays have been a major focus of biosensor research.

**Figure 14.**
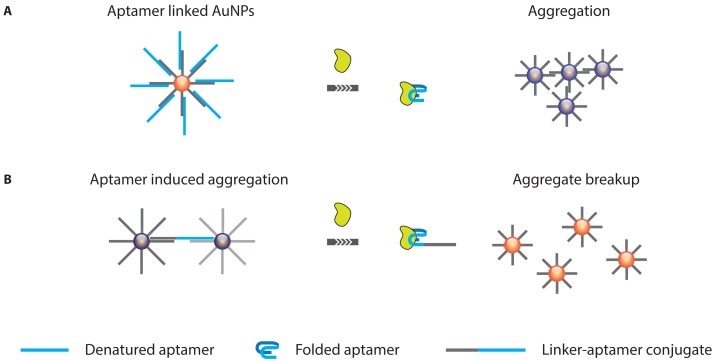
AuNP nanoparticle based protein detection strategies. (**A**) Target induced aptamer release resulting in AuNP aggregation. (**B**) Target induced aggregate breakup. Both approaches make use of the fact AuNP aggregation induces a coupling of their plasmon bands that leads to a color change from red to blue.

A large number of colorimetric aptamer bioassays rely on the unique optical properties of AuNPs. Suspensions of these nanometer-sized gold clusters typically display a range of deep-red colors due to visible light absorption in the range from 500 to 650 nm. When these particles get into close proximity of one another however, interactions between the plasmon bands of individual particles will cause the aggregates to turn blue with absorption of light above 650 nm. Since the color transitions described here are very obvious, even to the naked eye, AuNPs form the excellent basis for the creation of simple yet effective bioassays that in some cases do not even require any special readout equipment. Most of these assays are based on DNA functionalized AuNPs that can be made to aggregate in the presence or absence of analyte (Figure 14). Although specific implementations may differ, all of these assays rely on the fact that aptamers usually display significant conformational changes upon target binding [58]. These changes can prevent the aptamer from hybridizing with some form of partially complementary oligonucleotide. When this happens, the AuNP immobilized aptamer can no longer cause AuNP aggregation in the presence of AuNPs, functionalized with this complement or in other cases, e.g., when the complementary sequence also poses as a linker between two differently functionalized AuNPs, this event will actually cause aggregation (Figure 14[59–62]. Saha *et al.* [63] provided a very exhaustive overview of AuNP aggregation based colorimetric assays alongside other uses of AuNPs in biosensing.

Fluorescence based aptamer assays can broadly be divided into two groups; those that rely on a direct readout of the fluorescent label attached to aptamers and those that rely on the appearance or disappearance of a fluorescent signal upon target binding. These changes in fluorescence are usually caused by aptamer conformational changes in combination with some form of dual fluorophore-quencher labeling. In the case of direct fluorescence readout of labeled aptamers, it becomes essential to devise a strategy for differentiating bound from unbound aptamers. Since the fluorescence properties of the dye label usually will not change upon target binding, it is impossible to directly use the fluorescence signal for differentiation and quantification of bound and unbound states. Different solutions to this problem exist. One can attempt to separate target bound from unbound aptamers, e.g., using capillary electrophoresis (CE) [64]. Alternatively, the aptamer fluorescence can be used indirectly to readout another property that is affected by target binding, such as fluorescence anisotropy [65]. Only in a few exceptional cases will the dye label reside in very close proximity to the recognition site between an aptamer and its target or a site of conformational change [66], thus obviating the need for separation of bound and unbound aptamers.

Strategies that rely on more than one reporter have also proven to be practical in that they generally allow one to attempt optical signal transduction based on fluorescence resonance energy transfer (FRET). Here, target binding will introduce conformational changes. These will either bring a fluorescent label in close proximity to a quencher or a secondary fluorophore or move it away. This allows all kinds of turn on/turn off fluorescence assays to be developed [57].

Next to fluorescence based aptasensing strategies, a host of alternative optical strategies exist, such as (electro)chemiluminescence [67], aptazyme linked colorimetric assays [68] and many others. An excellent review on the large variety of existing optical methods is provided by [56,79].

#### 3.1.2. Mass Based Aptasensing Assays

Many examples exist where aptamers are used as the recognition element in mass based biosensors, particularly quartz crystal microbalance (QCM) [70–72] and surface plasmon resonance (SPR) [72–75] or even impedimetric formats [76]. Most of these assays rely directly on the change of mass at the sensor surface upon binding of the target by the aptamer. One of the general advantages offered by these methods is the lack of a need for specific labeling of the affinity aptamers or their targets. While this approach has proven feasible, even in the presence of complex sample matrices [77], the reported limits of detection are usually quite high [56,75,78] with only a few reports of assay performances that rival ELISA [79]. Therefore, the direct utility of these assays is limited to relatively large and more abundant analytes whereas small organic molecules or metabolites are usually unable to cause sufficiently large accumulation of mass at the surface.

**Figure 15.**
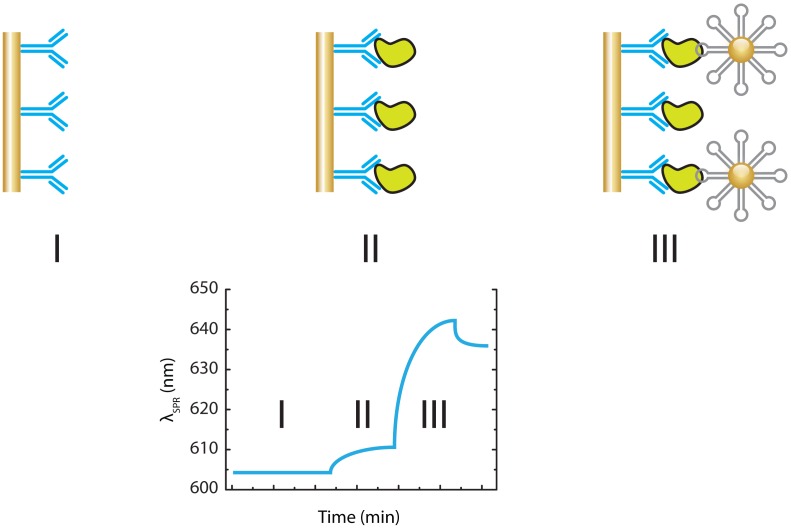
A schematic representation of the PCR sandwich immunoassay for the ultrasensitive detection of hIgE that utilizes AuNPs–aptamer conjugates as amplification reagent [80]. (**I**) The SPR sensor is functionalized with pAbs targeting the analyte. (**II**) The analyte of interest is captured on the sensor surface causing a slight shift of the SPR signal. (**III**) Aptamer functionalized AuNPs act as a secondary label. The high density of the AuNPs causes a significant amplification of the SPR signal.

In an effort to improve the limits of detection for aptamer based SPR, [80] explore the amplification effect of aptamer-AuNP conjugates for detection of large biomolecules. For this purpose they devised a sandwich immunoassay targeting human immunoglobulin E (hIgE) as model analyte. In the assay, hIgE, captured by immobilized goat anti-human IgE on an SPR gold film, is detected by SPR spectroscopy with a detection limit of 1 ng/mL when anti-hIgE aptamer-AuNP conjugates are used as signal amplification reagents. The authors also show that the non-specific adsorption of the aptamer-AuNPs conjugates on goat anti-hIgE is confirmed by SPR spectroscopy and then it is minimized by treating aptamer-AuNP conjugates with 6-mercapto-1-hexanol (MCH) (Figure 15).

### 3.2. Non-PCR Signal Amplification

The previous assays used various forms of labeled aptamers for the direct detection of the aptamer or target binding. Assay sensitivity therefore usually relied on the sensitivity of the transduction technology that was used, be it optics, SPR or any other technology. As will be discussed in the final paragraphs of this review, a number of innovative aptamer assays also make use of enzymatic amplification of aptamers or their label oligonucleotides upon target binding using PCR or RCA. However, more recently there have been a number of intriguing reports on aptamer based protein detection that make use of signal amplification using methods that do not rely on traditional PCR or even *de novo* DNA synthesis and for that reason they are particularly interesting. Indeed, their signal enhancement methods do not rely on the thermal cycling that is normally necessary for PCR, a requirement that might limit the implementation of PCR based assays e.g., on microfluidic or other miniaturized platforms.

**Figure 16.**
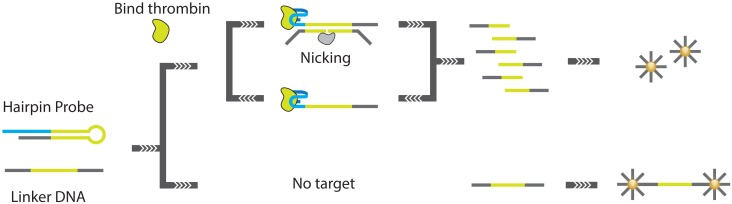
A schematic overview of the CESA [81].

Li *et al.* [81] report on a colorimetric assay for highly sensitive detection of not only proteins but also small molecules. The assay, which is called cyclic enzymatic signal amplification (CESA) relies on the use of a hairpin aptamer probe, a linker ssDNA, two sets of DNA-modified AuNPs, and nicking endonuclease (NEase). In the absence of target, the hairpin aptamer probe and linker DNA can stably coexist in solution. The free linker ssDNA can serve as a template for AuNP aggregation. However, in the presence target, the hairpin structure of aptamer probe is no longer favored and the probe-target complex will hybridize the linker ssDNA. Upon formation of this duplex, the NEase cleaves the linker DNA into two fragments and this process will repeat itself. The cleaved fragments of linker ssDNA are no longer able to cause AuNP aggregation, which will result in a color change of the AuNP solution. This simple method for signal amplification allows for a colorimetric interrogation of the sample where the amount of red-shift of the absorbance maximum is a direct measure for the amount of analyte present. In this way, the authors were able to report an LOD of 50 pM to 100 nM for protein and small molecule targets respectively, which marks a significant improvement over aptamer based assays targeting the same analytes but which do not use signal amplification. Some of the advantages of this method are the fact that NEase recognition sites do not need to be incorporated into the aptamer sequences themselves but instead can be incorporated into the linker, minimizing the risk of negatively influencing aptamer affinity and specificity. Drawbacks might be that the sensitivity of the assay is limited by the resolution of the spectroscopic equipment used for readout as well as the fact that the amplified signal will inevitably saturate after prolonged incubation times, which can also limit the resolution (Figure 16).

**Figure 17.**
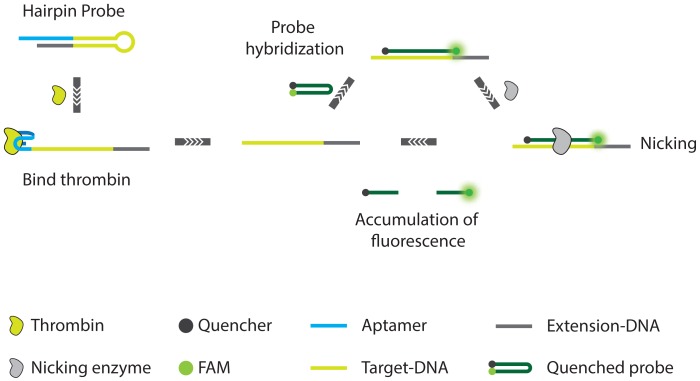
A schematic overview of the NEFSA assay. In the assay, an existing aptamer is extended with tailing sequences such that the free aptamer forms a hairpin structure. Target binding destabilizes this hairpin structure, which enables hybridization of a quenched fluorescence probe [82,83].

Similarly, Xue *et al.* [82,83] developed an aptamer nicking enzyme assisted fluorescence signal amplification (NEFSA) assay based on a hairpin probe and nicking enzyme assisted signal amplification. The metastable state hairpin probe with short loop and long stem was designed to contain a protein aptamer for target recognition. A short black hole quencher (BHQ) fluorescence DNA probe carrying the recognition sequence and cleavage site for the nicking enzyme is employed for fluorescence detection. Introduction of target protein into the assay leads to a conformational change of the hairpin probe, thus facilitating the hybridization of the BHQ probe. The fluorescence signal is amplified through continuous enzymatic cleavage. Using human *α*-thrombin as a model analyte, the assay displays a detection limit of 100 pM. Since the assay can be performed isothermally and without the need for immobilization of the affinity probes, the assay is suited for implementation on biosensing platforms (Figure 17).

**Figure 18.**
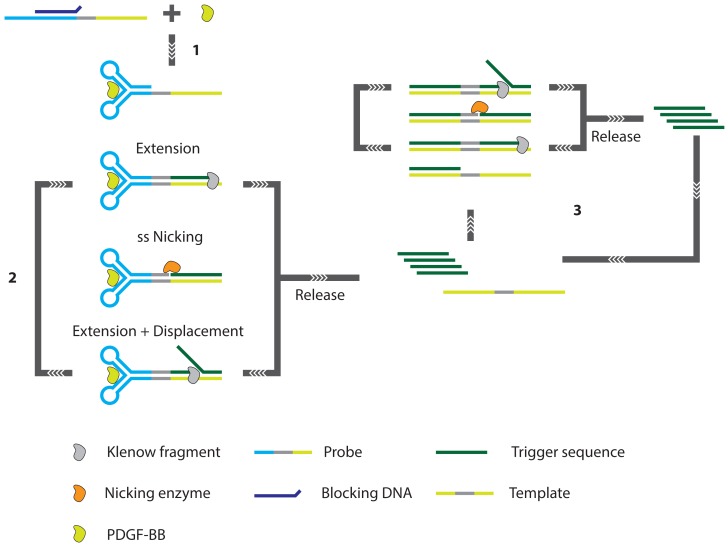
The exponential amplification reaction based assay involves three principal steps: (**1**) target-aptamer binding and blocker DNA dissociation, (**2**) linear strand displacement amplification, and (**3**) exponential amplification reaction. It is the binding of the target protein, PDGF-BB, that triggers a conformational change of the extended aptamer. This conformational change enables a cascaded amplification of the trigger sequence that ultimately results in a detectable signal [84].

Finally, Zhang *et al.* [84] demonstrate a strand displacement amplification (SDA) in combination with a two-stage exponential amplification reaction (EXPAR) (Figure 18). The assay makes use of an aptamer based probe that consists of three regions. The first region contains the actual aptamer used for target recognition. This aptamer is extended at the 5′ terminus with a recognition site for a single strand nicking enzyme and the template for the EXPAR. In the presence of a ssDNA blocking oligonucleotide, the aptamer cannot fold into its active conformation and a dangling end on the blocking oligonucleotide will prevent extension of its 3′ end such that the correct dsDNA substrate for the nicking enzyme cannot be created. However, when the correct target is present, aptamer folding is favored over hybridization of the blocking probe. An extensible duplex is formed and the resulting dsDNA can be correctly nicked. After nicking, the Klenow fragment enzyme can re-initiate dsDNA synthesis, displacing the previously formed strand. In this way, a cyclic process (the SDA) is started leading to a linear increase of ssDNA. Exponential amplification of DNA is achieved by a second SDA where the aptamer probe is replaced by a so-called EXPAR template, which is similar to the aptamer probe in all but the first sequence region where the aptamer is replaced with a sequence that is complementary to the product of the original SDA. Finally, the accumulation of newly created dsDNA can be visualized through the use of intercalating fluorescent dyes. This detection method exhibits excellent specificity and high sensitivity with a detection limit of 0.9 pM and a detection range of more than 5 orders of magnitude.

Moreover, this detection method has significant advantages in terms of isothermal conditions required, simple and rapid without multiple separation and washing steps, low-cost without the need of any labeled DNA probes. Furthermore, this method might be extended to sensitive detection of a variety of biomolecules whose aptamers undergo similar conformational changes.

### 3.3. PCR and RCA Enhanced Detection Aptamer Based Protein Detection

Since aptamers are nucleic acid oligomers, it is obvious that their numbers can be amplified in a sample using one of the many polymerase based methods that are available. This has led to the development of aptamer-only implementations of many of the previously discussed protein detection assays such as IPCR, PLA and PEA and in most cases, the absence of antibodies in the assay can lead to significant advantages.

In IPCR these advantages of using oligonucleotide aptamers are immediately apparent. Indeed, next to the generally more desirable stability and more straightforward production of aptamers over antibodies, the major technological challenge of IPCR, *i.e.*, coupling of the antibody to a DNA label, is much less problematic. This is exemplified in the work of Yoshida *et al.* [85] where the use of aptamers targeting IgG in an immunoassay makes labeling of the secondary antibodies through a direct link, be it biotin-streptavidin, covalent or otherwise, redundant. Instead, their approach relies on recognition of IgG by a specific aptamer. This way, in what is otherwise a straightforward sandwich type assay format, presence of target in the sample will ultimately lead to the binding of aptamer on the secondary antibody. This aptamer can be subsequently amplified using PCR, resulting in a quantitative readout [85]. In an alternative example of this assay, the IgG aptamer was extended with a dsDNA tag for use as a quantitative PCR (qPCR) template [86]. These assays still ultimately rely on the use of antibodies however and therefore share all drawbacks resulting from their use.

One of the earliest true examples of aptamer based IPCR even shows how antibodies can be completely avoided as Fischer *et al.* [87] extend an anti-human *α*-thrombin aptamer with a general linking sequence and a more specific extension that allows the aptamer to be primed for PCR amplification or to serve as a primer in RCA. This way, the extended aptamer is used in a sandwich assay using magnetic nanoparticles (MNPs) where it replaces the secondary antibody. After incubation, the aptamers bound to analyte on the MNPs are washed off and subjected to either PCR or RCA allowing for the quantification of target. By using two mechanisms of amplification, the authors are able to achieve a wide dynamic range for the assay. They show how PCR is more suited for quantification of very low levels of aptamer whereas the linear amplification offered by RCA makes the system inherently more suited for the quantification at higher levels of analyte. Similarly, Pinto *et al.* [88] very recently proposed a variation of the aptamer IPCR where photoswitchable, “caged” aptamers were used for the detection of human *α*-thrombin through an IPCR assay. Here, aptamer target affinity can be deactivated using light allowing more efficient and controllable aptamer release prior to PCR amplification.

A major issue encountered with aptamer IPCR is the occurence of non-specific interactions of the aptamers with the carriers (*i.e.*, MNPs or plate wells) used in the assay. This could lead to the presence of amplifiable DNA in the samples, even when no analyte was present. Yang and Ellington [89] and later Wu *et al.* [90] show how this problem can be circumvented by modifying the aptamer based IPCR to make use of the inherent structure switching abilities of many aptamers upon target binding (*vide supra*). In each of the examples, the authors devised an aptamer based assay targeting platelet derived growth factor (PDGF) or human *α*-thrombin where the sample is incubated together with aptamer. In the work of Yang and Ellington [89], this target binding will expose regions of the extended aptamer sequences that can be used for primer binding during PCR whereas Wu *et al.* [90] make use of a second ssDNA “copy DNA (cDNA)” sequence. In the absence of target or when aptamer is sorbed onto the walls of sample tubes or carriers, this cDNA will hybridize with the target. However, when the aptamer is effectively bound to target, folding of the aptamer will prevent any cDNA hybridization from occurring. When this happens, the cDNA can serve as a primer for the initial circularization and subsequent RCA of a third ssDNA sequence. This way, the authors show how quantification of target can be achieved while minimizing the risk of non-specific interactions from generating a false positive result.

**Figure 19.**
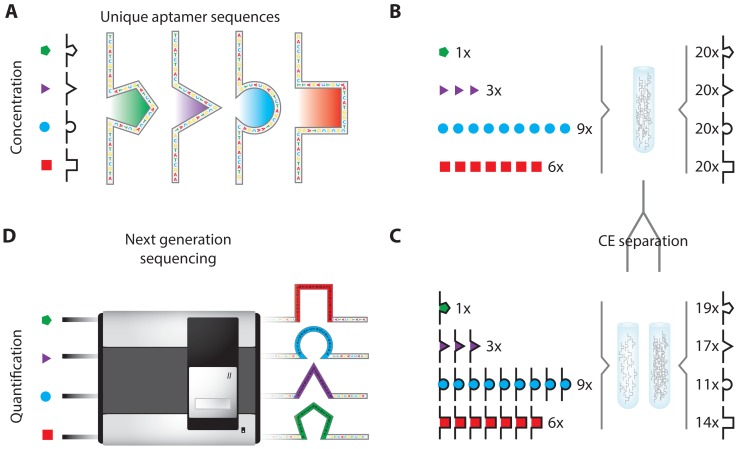
The application of NGS to protein diagnostics: An aptamer collection containing an excess of aptamers with established affinities against the analytes of interest (**A**) is used to incubate the sample under investigation (**B**). All different aptamer-analyte complexes are separated from the excess (unbound) aptamers (**C**) after which the bound aptamers are analyzed by NGS (**D**). The analytes of interest are then identified and quantified by decoding and counting the nucleotide sequences of their respective aptamers [94].

In another attempt to lower background signals in aptamer based IPCR, Zhang *et al.* [91] use affinity prope capillary electrophoresis (APCE) to separate target-bound aptamers from free aptamer in what is essentially an IPCR assay. Here, the sample is incubated with an aptamer that acts as both the affinity reagent as well as the oligonucleotide label. After APCE separation, the target bound aptamer is subjected to PCR and slab gel electrophoresis based quantification. While the authors could show impressive limits of detection, as low as 30 fM for the HIV reverse transcriptase 1 (HIV RT1), their approach is somewhat limited as gel based DNA detection allows only very crude quantification without the possibility to discriminate between the desired amplicon and any amplification byproducts or multiple aptamers in a parallelized assay. These issues were addressed by Janssen *et al.* [92,93] who showed that APCE separation, combined with quantitative PCR (qPCR) detection, can be used for the simultaneous, ultra-sensitive quantification of different proteins in a single sample. Careful design of extensions to the native aptamer sequences enabled qPCR for the detection of multiple aptamers in a mixture resulting from APCE without compromising the original aptamer target affinity. In another embodiment of aptamer APCE, Turner *et al.* [94] demonstrate the potential of massively parallel next-generation sequencing (NGS) for both target identification as well as quantification (Figure 19). These developments show how aptamer APCE has the potential to be applied for the multiplexed, ultra-sensitive analysis of large numbers of target proteins in complex sample matrices.

Finally, the fact that oligonucleotide aptamers are sensitive to enzymatic digestion by nucleases can also be used as an efficient method for background suppression in aptamer based IPCR. Wang *et al.* [95] demonstrate how the binding of antihuman *α*-thrombin DNA aptamer to its target succeeds in making the aptamer less sensitive to enzymatic degradation. This way, any aptamer that is non-specifically interacting with the sample or the sample container will be digested and only target bound aptamer will remain available for qPCR amplification, resulting in reduced background. Zheng *et al.* [96] have recently shown how this approach can be universally extended to multiple aptamer target pairs, even when dealing with small analyte molecules. Other interesting examples of aptamer IPCR include the work of Qiu *et al.* [97] who designed dual target oligonucleotides that include regions targeting proteins as well as specific DNA sequences that are relevant to disease marker detection.

The earliest examples of PLA actually did not involve antibody-DNA adducts but instead relied on the use of multiple aptamers against a single target [42]. Aptamer based PLA naturally offers all advantages that were previously discussed in relation to antibody based variations of the assay, namely a homogeneous assay format and background suppression as the likelihood of proximity ligation in the absence of analyte is extremely low.

Aptamer based PLA was modified to use RCA for signal amplification. For this purpose, Di Giusto and King [98] designed circular aptamers, so-called “captamers”, which, aside from improved stability against enzymatic degradation and affinity/avidity, can be used as template for RCA. In a proof of concept study, Di Giusto and King [98] show how a captamer targeting human *α*-thrombin is combined with a second, linear aptamer targeting the same protein. Upon simultaneous binding, the linear aptamer can serve as a primer for the RCA captamer. As such, the aptamer combines RCA with PLA and the prospect of combining the signal amplification abilities of RCA with multifunctional captamers offers some interesting opportunities for the future development of multiplexed assays.

Next to the obvious use of RCA as a signal amplification method, it also allows the incorporation of additional functionality into the amplified material. When a sequence, complementary to DNAzyme is present in the circular RCA template, the amplicon will display enzymatic behavior, e.g., horse radish peroxidase (HRP) activity, which will enable simple colorimetric identification of the amplicon [99]. The same authors also show how multiple aptamers can be combined in a single RCA amplicon, giving rise to the potential for simultaneous detection of multiple targets [100]. Other possibilities include the incorporation of sites that have the ability to modulate the fluorescence behaviour of specific dyes upon target binding within the RCA product [101].

## 4. Conclusions

It is clear that the use of DNA labeling can serve to significantly enhance the sensitivity of immunoassays for the detection of protein biomarkers. With the development of IPCR [18], it became possible to analyze concentrations of important markers at levels that were orders of magnitude below what was possible with conventional ELISA. Even though the work of Sano *et al.* [18] constituted a major advancement, significant effort has since been directed towards making IPCR more accessible, e.g., with the development of methods to make the production of oligonucleotide labeled antibodies more straightforward [21,24]. Alternative DNA amplification strategies such as RCA were leveraged to unite IPCR with the practical requirements for multiplexed, array based detection, enabling high throughput applications [36–39]. Bio-barcode assays [30] and magneto IPCR offered the possibility of sample concentration and background removal. In this respect, the development of PLA and PEA can also be viewed as evolutions of IPCR technology that offer enhanced resistance to experimental noise and cross-reactivity often experienced when targeting multiple analytes in clinical samples.

Even though the use of DNA antibody labeling technology has matured to provide high sensitivity immunoassays with excellent scalability towards multi-analyte screening and complex sample matrices [36], the use of more easily produced oligonucleotide affinity reagents, *i.e.*, aptamers, could help to further advance the development and use of ultra-sensitive protein assays. Indeed, oligonucleotides are easy to produce at scale, can be extensively modified and offer significant structural variability and even advanced structure switching behaviours [56], which can all be leveraged in innovative and novel assay formats.

While examples of aptamer based assays and biosensors now exist, often with performance characteristics that can rival the state-of-the-art in oligonucleotide labeled immuno assays, significant challenges remain before the use of aptamers can become commonplace in clinical or even research analytical settings. For one, the ability to identify new aptamers against relevant targets, be they proteins or small molecules, has long been a limiting factor. Most reports on aptamer technology focus on only a handful of known aptamers and relatively little effort is directed towards the selection and development of novel aptamers [102]. To make matters worse, many reports on aptamer based biosensors even use specific or even unique characteristics of this relatively small group of known aptamers (e.g., folding) as a key element of an assay, which can stand to severely limit the universal nature of some of these aptamer biosensors.

Recently however, development of new olignucleotide chemistries have shown promise for enabling more rapid aptamer discovery [103]. Importantly, these new classes of aptamers can also help to provide a solution for another major problem confronting aptamer based biosensing—nucleic acid degradation due to e.g., serum nucleases [98,103]—while still maintaining compatibility with common enzymatic oligonucleotide replication [104]. These developments, in combination with automated and highly sophisticated selection techniques [105–108], might help to improve the properties of newly discovered aptamers and the acceleration of their adoption in clinical sensors [109].

Finally, with the ever increasing availability of sensitive protein detection strategies and high affinity immuno-reagents, be they antibodies or aptamers, research groups are steadily striving to apply these novel technologies towards a common ultimate goal: the discovery of blood or plasma protein biomarkers for disease detection. In this respect, recent literature already provides some impressive examples showing how the multiplexed detection of various proteins can be used for the identification of various pathologies such as cardiovascular disease [51], chronic kidney disease [103] or lung cancer [110]. Despite these initial efforts and despite the existence of large numbers of biomarker candidates in the human proteome, identification and successful application of new biomarkers in a clinical context remains highly elusive [111]. The causes for these difficulties are various [112]. First of all, the human population is intrinsically highly diverse and protein expression levels may vary with age, gender or genetic descent of an individual. Secondly, specific pathologies, such as cancer, might cause protein expression levels to change significantly in relation to disease progression or even in the area of the body that is affected. Finally, blood and serum, the matrices in which biomarkers often need to be detected, are very complex media that contain dozens of highly abundant proteins that can interfere with the specific and sensitive detection of the analyte of interest. In addition, preparative steps such as blood sampling, centrifugation or sample storage might also cause unpredictable effects such as platelet- or leukocyte lysis as well as coagulation to occur, further interfering with detection. Brody *et al.* [112], Mehan *et al.* [113] as well as Landegren *et al.* [114] provide excellent discussions of these phenomena in relation to highly multiplexed protein diagnostics. If one conclusion should be drawn here, it would be that there are limits to what can be achieved through the search for ever more sensitive affinity reagents and assays. In a complex sample matrix containing background proteins at concentrations that might be many orders of magnitude higher than that of the analyte of interest, non-specific interactions can and most often will occur. As such, one cannot rely on equilibrium binding alone to achieve biomarker identification and detection [115]. New strategies have to be developed to enhance the specificity in multiplexed assays, either through reliance on multiple distinct binding events [51] or through kinetic distinction between non-specific and specific interactions [112,115]. Only when these strategies are combined with new powerful bioinformatics and statistics methods [112] will robust biomarker based disease detection become a reality.
